# PPDiffuse: A Quantitative Prediction Tool for Diffusion of Charged Polymers in a Nanopore

**DOI:** 10.6028/jres.125.018

**Published:** 2020-06-19

**Authors:** David P. Hoogerheide

**Affiliations:** 1 National Institute of Standards and Technology, Gaithersburg, MD 20899, USA

**Keywords:** biopolymers, calculator, drift-diffusion, first-passage time, nanopore, polynucleic acids, polypeptides, smoluchowski equation

**Software DOI:** https://doi.org/10.18434/M32176

**Software Version:** 1.0

## 1 Summary

**Fig. 1:**
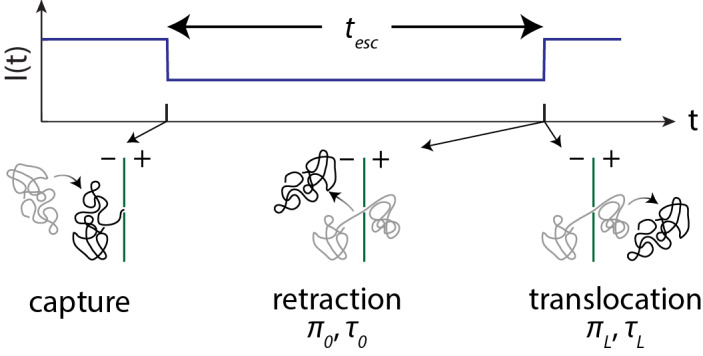
A typical nanopore-based biopolymer sensing experiment.

Nanopore-based sensing of charged biopolymers is a powerful single-molecule method. In a conventional nanopore experiment, a single biological (proteinaceous) or solid-state nanopore perforates a thin membrane that is wetted by, and electrically isolates, two opposing reservoirs of electrolyte solution. A potential is applied across the membrane via external electronics coupled to the electrolyte reservoirs with electrochemical electrodes, actuating the system. The electric field set up by the applied potential in the nanopore and its immediate environment plays two roles: supporting an ionic current through the nanopore, which reports on the properties of the pore and its contents; and acting on analyte molecules to attract them to, and drive them into, the nanopore. The presence of a large biopolymer in the pore modulates the ionic current I(t). The duration of the ionic current modulation corresponds to the length of time the polymer spends in the pore from capture to its ultimate escape, either by retraction to the reservoir from which it was captured, or by translocation to the opposite reservoir (Fig. 1). The probabilities of retraction or translocation, or splitting probabilities, and the corresponding distributions of escape times ($t_{esc}$), are particularly sensitive to the size and charge of the analyte molecule and have been the focus of much theoretical, computational, and experimental effort.

For homogeneously charged, linear biopolymers such as DNA, which do not interact strongly with the surface of the membrane, the physical basis of the escape time is intuitive. For most experiments, the electrical driving force required to capture DNA molecules into a nanopore is large enough to overcome thermal diffusive motion. Thus, the translocation process dominates, and larger potentials result in a larger driving force and shorter escape times.

In the case where the polymer interacts with the membrane, or is heterogeneously charged, as is generally the case for nanopore capture of polypeptides, this intuition fails. As the polypeptide moves through a nanopore, the direction of the electrical force from a constant applied potential varies with the sign of the charge of the amino acids in the channel; in addition, membrane interactions provide a retarding force that can either neutralize or enhance the electrical effects. In this milieu, thermal diffusive forces can dominate the dynamical motion of the polymer in the pore.

An underlying physical framework in which the distribution of escape times is modeled as a first-passage time from a one-dimensional potential is quantitatively predictive for a wide range of experiments [1-4]. The complexity of this potential for the general case, however, requires calculations to guide experimental design that can be tedious to implement. *PPDiffuse* is intended to remove this burden from the nanopore research community and enable convenient, rational design of nanopore experiments with complex substrates such as polypeptides.

*PPDiffuse* is written as a one-page web application using Javascript hosted at https://pages.nist.gov/ppdiffuse/ppdiffuse.html, with extended instructions for use at https://pages.nist.gov/ppdiffuse.

## 2 Software Overview

### 2.1 Calculation Overview

The dynamics of motion are described by a one-dimensional Smoluchowski (drift-diffusion) equation for diffusion in an one-dimensional interaction potential $U\left(x\right)$ [5]. The reduction in dimensionality from three to one arises from the linear nature of the polymer; the length of polymer that has passed through the nanopore is the single spatial dimension $x\in \left(0,L\right)$, where L is the contour length of the polymer. The distribution of event durations is modeled as the first-passage time to the boundaries of the spatial domain, which correspond to escape of the polymer from the nanopore. Arrival at the x=0 and x=L boundaries correspond to retraction and translocation process, respectively. The basic function of *PPDiffuse* is to construct $U\left(x\right)$from physically relevant inputs and calculate the corresponding splitting probabilities and first passage times. These are the zeroth and first moments of the distributions of retraction and translocation escape times and can be expressed analytically as spatial integrations over $U\left(x\right)$ [6]. The quantities calculated here are the probability of retraction $\pi_{0}$, the probability of translocation $\pi_{L}$, the conditional average escape time for retraction $\tau_{0}$, the conditional average escape time for translocation $\tau_{L}$, and the average escape time $\tau =\pi_{0}\tau_{0}+\pi_{L}\tau_{L}$. For tethered polymers (Sec. 2.2.2), the probability of translocation is zero by construction, so only the average escape time and the injection point positions are reported.

### 2.2 Calculation Inputs

Calculation of the splitting probabilities and first passage times requires three inputs. The first is the effective diffusion constant, which describes the mobility of the polypeptide chain in the channel and is typically (0.1 to 10) µm^2^/s. While in principle the diffusion constant can be position-dependent, in *PPDiffuse* it is assumed constant. The second input is the “injection point” $x_{0}$, which corresponds to the initial position of the system. *PPDiffuse* determines the minimum of the potential within a user-defined range to be the most stable initial position and hence the injection point. In practice, this range can be made arbitrarily small and the injection point thereby forced to a fixed position.

The third input is the interaction potential $U\left(x\right)$, which comprises all the quasi-conservative physical and statistical forces acting on a biopolymer in a nanopore. This calculation includes (1) electrokinetic forces; (2) entropic forces; and (3) other generalized forces. Each is described in the following; the total interaction potential function is simply the sum of all the interaction terms.

#### 2.2.1 Electrokinetic Forces

Electrokinetic forces arise from the action of the applied electric field on the charges in the nanopore. These include direct interactions between the electric field and the charges on the polypeptide, which depends only on the amino acid sequence of the polypeptide; as well as hydrodynamic drag from the electroosmotic flow (EOF) in the nanopore, which depends to a first approximation on the total fixed charge in the nanopore (polypeptide amino acids plus charge on the pore walls). The expression for the electrokinetic force is

**Table:** 

	$U_{E}\left(x\right)=V\int_{0}^{x}\sigma \left(x՛\right)dx՛,$	(1)

where V is the applied transmembrane potential and $\sigma \left(x\right)$ is the effective charge density. The effective charge density is the native charge density $\sigma_{n}\left(x\right)$ corrected for EOF. Because EOF is also linear in V, the simplest correction is linear: $\sigma \left(x\right)=m_{EOF}\sigma_{n}\left(x\right)+b_{EOF}$. The intercept $b_{EOF}$ is a correction for the drag arising from EOF from any charges on the pore walls; for a pore with an electrically neutral lumen, this term is expected to be zero. The slope $m_{EOF}$ is the reduction in effective charge density due to EOF from the charges on the polypeptide itself and is essentially a geometric factor. For solid-state pores, $m_{EOF}$ can be as low as 0.1 [7]. For biological pores, it is generally closer to unity. The default values, $m_{EOF}=0.654$ and $b_{EOF}=-0.21e^{-}/nm$, were estimated for the voltage-dependent anion channel (VDAC) from published results using the α-synuclein protein [2, 4].

The native charge density is calculated from the amino acid sequence of the analyte polypeptide, as provided by the user, and smoothed by the effective length of the nanopore. The expression is

**Table:** 

	$\sigma_{n}(x)=\sum_{i=1}^{N}z_{1}eA_{i}^{-1}\exp\left(\frac{-{\left(x-\left(i-0.5\right)l_{aa}\right)}^{2}}{2\sigma_{p}^{2}}\right)$	(2)

where $z_{i}$ is the charge number (+1, 0, or ‒1) of the *i*th of N total amino acids, *e* is the elementary charge, $\sigma_{p}$ is the standard deviation of a Gaussian distribution with full width at half maximum equal to the pore length, and $l_{aa}$ is the distance between amino acids and defaults to 0.4 nm [8]. The normalization factor $A_{i}$ ensures that the total charge of the *i*th residue is $z_{i}e$, particularly near the edges of the potential where the Gaussian distributions are truncated. This procedure preserves the total charge of the polypeptide. The expression is

**Table:** 

	$A_{i}=\int_{0}^{L}\exp\left(\frac{-{\left(x-\left(i-0.5\right)l_{aa}\right)}^{2}}{2\sigma_{p}^{2}}\right)dx.$	(3)

The direction of the amino acid sequence also matters, because the amine group at the N-terminus contributes an additional positive charge, while the carboxyl group at the C-terminus contributes an additional negative charge. Thus, $z_{1}$ is automatically incremented by 1, while $z_{N}$ is decremented by 1.

The user-defined inputs are therefore the amino acid sequence, its direction (N- to C-terminus, or C- to N-terminus), the length per amino acid $l_{aa}$, the pore length $L_{p}\approx 2.355\sigma_{p}$, the electroosmotic slope and intercept parameters $m_{EOF}$ and $b_{EOF}$, and the applied transmembrane potential V. The amino acid sequence is represented as a string of single-letter amino acid codes (white space is ignored). Amino acid codes “D” and “E” have $z_{i}=-1$; “H”, “K”, “R” have $z_{i}=+1$; and all others have $z_{i}=0$. Double-stranded nucleic acid bases, or phosphorylated residues, which have $z_{i}=-2$, are represented by “X”.

#### 2.2.2 Entropic Forces

The mean first passage times calculated here are statistical quantities, *i.e.* averages over many assumed realizations or trajectories of motion in the interaction potential. As such, entropic terms can be calculated that account for the likelihood of the various states that can be adopted by the polypeptide during its tenure in the nanopore. For diagnostic purposes, the entropy term can be turned off by unchecking the “Use entropy?” checkbox.

The functional form of the entropic term depends on whether the ends are free or tethered. If both ends are free, the expression is

**Table:** 

	$U_{S}\left(x\right)=\nu k_{B}T\left[\ln\frac{x}{L}+\ln\left(1-\frac{x}{L}\right)\right].$	(4)

Here the Flory exponent ν is fixed at 0.59, the value for a self-avoiding Gaussian chain.

If, on the other hand, one end is tethered, the expression is more complex. The derivation is given in Ref. [9] and uses a non-self-avoiding random Gaussian chain for simplicity. The Kuhn length b defaults to 0.6 nm [8]. For an arbitrarily small distance δ (fixed at $10^{-3}\;nm$); a number of Kuhn lengths $n_{T}=\left(L-x-L_{p}/2\right)/b$ that have not translocated and a number of Kuhn lengths $n_{C}=\left(x-L_{p}/2\right)/b$ that have; a pore length $L_{p}$; and a tethering distance above the pore $l_{t}$, the entropic expression (to within an additive constant) is

**Table:** 

	$U_{S}\left(x\right)=-k_{B}T\ln\left[{\left(\frac{2\pi n_{C}b^{2}}{3}\right)}^{-\frac{3}{2}}\left[\exp\left(\frac{-3{\left(l_{t}-\delta \right)}^{2}}{2n_{C}b^{2}}\right)-\exp\left(\frac{-3{\left(l_{t}+\delta \right)}^{2}}{2n_{C}b^{2}}\right)\right]\mathrm{erf}\left(\sqrt{\frac{3}{2n_{T}}}\frac{\delta }{b}\right)\right].$	(5)

The user inputs are the pore length $L_{p}$ (common to the electrokinetic term in Sec. 2.2.1), the polymer length L (derived from the amino acid sequence), whether the polymer is free or tethered, and, only if tethered, the distance $l_{t}$ between the tethering point and the pore and the Kuhn length b.

#### 2.2.3 Other Interactions

*PPDiffuse* allows addition of an arbitrary number of additional interaction terms. Currently three functional forms of these terms are allowed, though they are incorporated in a modular way so that additional functional forms can be added in a straightforward manner. These forms are listed in the following sections with comments on their physical interpretation.

*Constant force.* A constant force term has the functional form $U_{F}\left(x\right)=Fx$. This term applies in situations where hydrodynamic or osmotic pressure exists over the membrane, creating hydrodynamic drag, as in Ref. [3].

*Gaussian potential.* A Gaussian potential has the functional form

**Table:** 

	$U_{G}\left(x\right)=E_{G}\exp\left(\frac{-{\left(x-x_{G}\right)}^{2}}{2\sigma_{G}^{2}}\right).$	(6)

It corresponds to non-voltage-dependent potentials that correspond to a particular location on the polypeptide, such as a bulky side chain that introduces an entropic potential ($E_{G}>0$), as in Ref. [4], or a site-specific interaction with the channel interior ($E_{G}<0$).

*Barrier potential.* A barrier potential has the functional form

**Table:** 

	$U_{B}\left(x\right)=E_{B}\mathrm{erf}\left(\frac{x-x_{B}}{\sigma_{B}\sqrt{2}}\right).$	(7)

This form was successfully used [2] to describe the energetics of the N-terminal binding region of the α-synuclein molecule. Barrier potentials of equal and opposite magnitude can be added to create smoothed “box function” potentials.

### 2.3 Graphical Interface

**Fig. 2:**
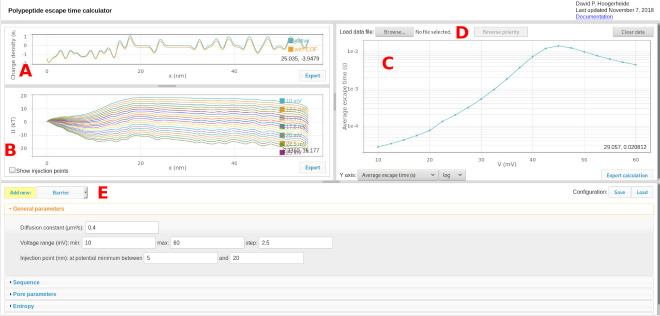
Screenshot of the one-page web application. Bold, red letters are used to reference the various features in the text.

The user interface for *PPDiffuse* is shown in Fig. 2. The bold, red letters label the different sections of the user interface, as follows:

A. The charge density plot displays the native charge density ($\sigma_{n}\left(x\right)$, labeled “native”) and the effective charge density ($\sigma \left(x\right)$, labeled “with EOF”), in units of $e^{-}/nm$. See Sec. 2.2.1.
B. The interaction potential plot displays the calculated interaction potential $U\left(x\right)$ for each specified transmembrane potential, in units of $k_{B}T$. The “show injection points” checkbox displays the injection points at each voltage and allows graphical manipulation of the range over which the injection points are determined. See Sec. 2.2.
C. The results plot displays the results of the first passage time calculations. For free (untethered) polymers, six quantities can be displayed and selected using the “Y axis:” dropdown: the average escape time τ, the conditional average escape time for retraction $\tau_{0}$, the conditional average escape time for translocation $\tau_{L}$, the probability of retraction $\pi_{0}$, the probability of translocation $\pi_{L}$, and the injection point positions. For tethered polymers, the probability of translocation is zero by construction, so only the average escape time and the injection point positions are reported.
D. A two- or three-column text data file (voltage, average escape time, and optional escape time uncertainty) can be loaded using the “Load data file:” control and will be plotted in the same graph as the calculation result. For files with multiple voltage polarities, the polarity can be reversed using the “Reverse polarity” button. Data are cleared using the “Clear data” button or by loading a new data set.
E. The parameters pane contains all the inputs detailed in Sec. 2.2. The non-optional inputs are grouped according to general calculation parameters (transmembrane potentials, diffusion constant, injection points), sequence (polymer properties such as amino acid sequence, sequence direction, length per monomer, and Kuhn length), pore parameters (length and electroosmotic parameters), and entropic parameters (whether to use the entropic term, whether or not the polymer is tethered, and, if so, the distance from the pore to the tethering point). Additional forces of the constant force, Gaussian, or barrier form can be added using the button and dropdown menu in the upper left. Each introduces its own set of parameters and the option to delete the object. The entire configuration can be saved to a JSON file or reloaded using the controls in the upper right.

### 2.4 Calculation Outputs

In addition to the graphical output, each of the graphical panes has an “export” button that will export its contents as a tab-separated text file. The format of the results plot, in particular, is compatible with its import function so that a particular calculation can be exported, re-imported, and compared to variant calculations.

## 3 Software Specifications

**Table:** 

**NIST Operating Unit(s)**	NIST Center for Neutron Research, Condensed Matter Science Group
**Category**	First-passage time calculator (Smoluchowski equation)
**Targeted Users**	Nanopore-based biosensor researchers
**Operating System(s)**	All (requires up-to-date web browser)
**Programming Language**	ECMAScript5 and HTML5
**Inputs/Outputs**	See Secs. 2.2 and 2.4
**Documentation**	https://pages.nist.gov/ppdiffuse
**Accessibility**	N/A
**Disclaimer**	https://www.nist.gov/director/licensing

## 4 Software Libraries

External, open source libraries used to build this application include JQuery 1.11.1, JQuery UI 1.12.1, JQuery Layout 1.4.4, and MathJS 4.1.1.

Plots were constructed using an implementation of D3 for scientific plotting [10, 11].

## 5 Methods for Validation

Because *PPDiffuse* is intended for aid in experimental design, calculation speed is prioritized over accuracy. Experimental measurement repeatability is often in the tens of percent, so single-digit percent computation errors are acceptable. Two special cases with analytical solutions are presented first to show the calculation accuracy; the last case compares *PPDiffuse* output to a previously published result (which itself is subject to computational inaccuracies), showing acceptable agreement.

### 5.1 Untethered Polymer, No Forces

The first passage time calculations do not have a general analytical solution, nor are there standard codes for calculating their result. In the special case where $U\left(x\right)=0$, however, there are simple analytical forms. The results are, using the notation from Sec. 2.1,

**Table:** 

	$\pi_{0}=1-\frac{x_{0}}{L}$	(8a)
	$\pi_{L}=\frac{x_{0}}{L}$	(8b)
	$\tau_{0}=\frac{L^{2}}{2D}\frac{\frac{x_{0}}{L}\left(2-\frac{x_{0}}{L}\right)}{3}$	(8c)
	$\tau_{L}=\frac{L^{2}}{2D}\frac{\left(1-\frac{x_{0}}{L}\right)\left(1+\frac{x_{0}}{L}\right)}{3}$	(8d)
	$\tau =\frac{L^{2}}{2D}\frac{x_{0}}{L}\left(1-\frac{x_{0}}{L}\right).$	(8e)

In *PPDiffuse*, the case $U\left(x\right)=0$ can be realized as follows. The sequence is set to an arbitrary sequence of 100 amino acids. The length per amino acid is set to 1 nm, so that L=100 nm. The entropy term is turned off. The effect of charge is turned off by setting both the pore EOF slope and intercept to 0.

**Fig. 3:**
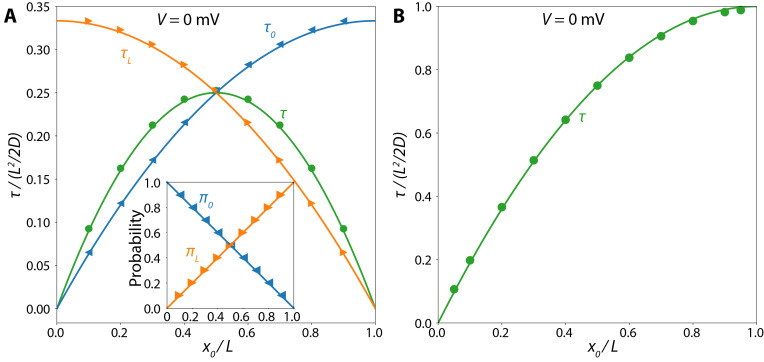
Comparison of first passage time analytical solutions (solid lines) to calculation results (points) for $U\left(x\right)=0$ and varying injection points $x_{0}$. (A) Mean first passage time (τ) and conditional first passage times for retraction ($\tau_{0}$) and translocation ($\tau_{L}$) processes of an untethered polymer diffusing in a nanopore in the absence of net force. (Inset) Splitting probabilities between retraction ($\pi_{0}$) and translocation ($\pi_{L}$). (B) Mean first passage time (τ) for a tethered polymer diffusing in a nanopore without net force.

For the calculation, the diffusion constant is set to $1{nm}^{2}/s$, and each of the outputs is calculated for a series of $x_{0}/L$. The results are plotted in Fig. 3A and show excellent agreement with the analytical expressions. Errors on the order of 1% are introduced by the numerical integration procedure. These can be improved by increasing the point density (fixed at $l_{aa}/2$) of the integration, but at the cost of speed.

### 5.2 Tethered Polymer, No Forces

To validate the computation engine for the tethered diffusion case, a similar procedure is employed. The only difference in the inputs is that the “Polypeptide tethered?” checkbox is selected. Because the entropy term is turned off, the “Tether length” field has no effect. The analytical solution for the mean first passage time to the x=0 boundary in this case is

**Table:** 

	$\tau =\frac{L^{2}}{2D}\frac{x_{0}}{L}\left(2-\frac{x_{0}}{L}\right).$	(9)

A comparison between the calculated results and the analytical solution is shown in Fig. 3B.

### 5.3 Free Diffusion, Constant Charge Density

**Fig. 4:**
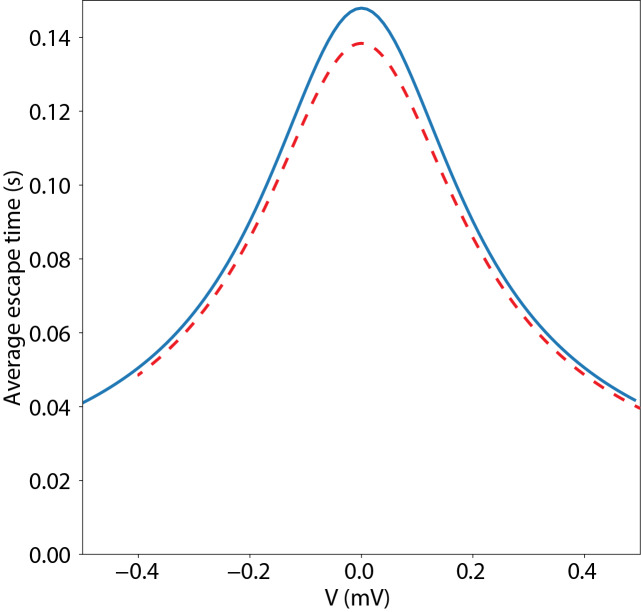
Comparison of calculated escape time (solid line) for 10 kilo-base pair double-stranded DNA in a solid state nanopore with previously published results (dashed line).

A more complex case including both a nonzero charge density and the free diffusion entropy can be constructed for comparison with Ref. [1]. In this case the diffusion of 10 kilo-base pair (L=3400nm) double-stranded DNA is studied in a solid-state nanopore. The diffusion constant was measured to be $7.10\;\mu m^{2}/s$, while the charge density was $0.275e^{-}/nm$. This situation could be modeled in *PPDiffuse* by using 10 000 base pairs, but more simply we use the sequence A(E)_98_X, which gives 100 charged units. We set the length per residue to 34 nm and adjust $m_{EOF}=9.35$ to give the correct charge density. The result of this calculation is shown in Fig. 4 as the solid curve; the dashed curve is reproduced from Fig. 2d of Ref. [1]. Of the small (5-10%) discrepancy, about half is due to numerical integration, and about half due to the implementation of the injection points, which are treated as Gaussian distributions in Ref. [1] but as delta functions in *PPDiffuse*. For the purposes of calculation in support of experimental design and initial modeling of experimental results, this difference is acceptable. Importantly, this calculation includes both the entropic function and a nonzero charge density, which determines the peak width, suggesting that both elements of the calculation engine are functioning correctly.
